# Associations between an *MDM2* gene polymorphism and ulcerative colitis by ARMS-PCR

**DOI:** 10.5808/GI.2020.18.1.e9

**Published:** 2020-03-31

**Authors:** Mahsa Sadat Hashemi Doulabi, Reza Goleyjani Moghaddam, Ali Salehzadeh

**Affiliations:** 1 Young Researchers and Elite Club, Tonekabon Branch, Islamic Azad University, Tonekabon 46841-61167, Iran; 2 Department of Biology, Tonekabon Branch, Islamic Azad University, Tonekabon 46841-61167, Iran; 3 Department of Biology, Rasht Branch, Islamic Azad University, Rasht 41476-54919, Iran

**Keywords:** inflammatory bowel disease, malignant, *MDM2*, polymorphism, ulcerative colitis

## Abstract

Ulcerative colitis is a form of inflammatory bowel disease characterized by chronic inflammation of the colon and rectum. The abnormal lesions in the digestive system caused by ulcerative colitis and intermittent colitis are of major clinical importance. MDM2 is a phospho-protein that functions as a ubiquitin ligase for p53. Recently, a T>G substitution in the promoter of the *MDM2* gene (rs309) has been identified. In this case-control study, 174 ulcerative colitis biopsy samples and 82 control samples were collected from colonoscopy centers, hospitals, and clinics in Mazandaran and Gilan Provinces in Iran from October 2014 to May 2015. This *MDM2* polymorphism was investigated in DNA samples (extracted from biopsy samples) by amplification-refractory mutation system polymerase chain reaction. The mean age of patients with ulcerative colitis was 46.5 years (range, 28 to 69 years) and that of control individuals was 45.3 years (range, 26 to 71 years). Seventy-eight patients (44.82%) were men and 96 (55.18%) were women. The distribution of the TT, TG, and GG genotypes was 17.93%, 27.59%, and 34.48%, respectively, in the ulcerative colitis patients and 31.70%, 24.40%, and 43.90%, respectively, in the control individuals (odds ratio of GG for ulcerative colitis, 7.142; 95% confidence interval, 2.400 to 9.542; p = 0.001). It was found that a single-nucleotide polymorphism at rs309 in the *MDM2* gene was associated with ulcerative colitis. A direct relationship was found between age and ulcerative colitis, while no relationship was found with sex. This finding is of note because the occurrence of intestinal inflammation and subsequent ulcers can precede the development of cancer.

## Introduction

Inflammatory bowel disease (IBD) is a complex disease that results from an inappropriate immune system response to intestinal bacteria [1]. IBD is generally subdivided into Crohn disease and ulcerative colitis . Ulcerative colitis is characterized by chronic inflammation of the colon and rectum, whereas Crohn disease can affect the entire digestive system. The role of genetic factors in these conditions was first raised by epidemiological studies that reported familial associations of these diseases [2].

Ulcerative colitis is a chronic inflammatory disease that affects the entire colon [3]. In ulcerative colitis, inflammation is classically confined to the colon, is typically persistent, and begins in the rectum [4]. The presence of abnormal gastrointestinal lesions in patients with ulcerative colitis and intermittent colitis is of major clinical importance, and many gastroenterologists therefore perform upper endoscopy to obtain a definitive diagnosis in patients with IBD [5].

The risk factors for ulcerative colitis appear to be related to changes in the intestinal microbiome or disorders in the intestinal mucosa [6,7]. Intestinal infections, non-steroidal anti-inflammatory drugs, and antibiotics all contribute to the development of IBD [7,8].

MDM2 is a phospho-protein and a ubiquitin ligase for p53 that is responsible for inhibiting p53 activity and promoting its destruction [9]. Recently, a T>G substitution in the *MDM2* gene promoter (rs309) has been identified. This substitution is associated with increased expression of *MDM2*, which accelerates the formation of several types of tumors, resulting in a tendency for them to occur at a younger age [10]. These findings underscore the importance of this polymorphism as an important factor that can affect the frequency of cancer in a population, the age of cancer in individuals, and individuals’ responses to cancer treatment [11].

The rs309 locus in the second promoter region of the *MDM2* gene, which is associated with increased expression of this gene, may have potential as a molecular target for cancer susceptibility and as suitable tumor marker. If a polymorphism is present at the rs309 position of the *MDM2* gene (i.e., a T>G conversion in this promoter region), the binding affinity of the SP1 transcription factor activator to this region is significantly increased, which increases *MDM2* gene expression. This means that an individual with a TT genotype for this polymorphism has a baseline expression level of the *MDM2* gene, but in an individual with the TG genotype, the G allele increases *MDM2* gene expression, and this expression is even more dramatically increased in individuals with a GG phenotype. Due to the inhibitory effect of MDM2 on p53, an increase in *MDM2* expression leads to a decrease in the intracellular amount of p53 protein, which is a key regulator of the response to cellular damage. Under usual circumstances, levels of the p53 protein are increased 5- to 14-fold when cellular damage occurs, but they are reduced by 2 to 3 times if the G allele is present in the rs309 locus of *MDM2*, which leads to increased levels of the MDM2 protein [12]. The overall aim of this study was to investigate the rs309 polymorphism of *MDM2* and its association with ulcerative colitis, and a secondary aim was to explore the association between this polymorphism and the risk of cancer.

## Methods

### Sample selection method

In this case-control study, 174 ulcerative colitis biopsy samples and 82 control samples were collected from colonoscopy centers, hospitals, and clinics in Mazandaran and Gilan Provinces, Iran from October 2014 to May 2015. Patients’ history, including age, sex, place of residence, and severity of illness was obtained, the diagnosis was confirmed by the treating physician, and a consent form and questionnaire were obtained. Then, in the colonoscopy procedure, some of the intestinal tissue was removed, transferred to sterile vials, and stored at –20℃ until DNA extraction.

### *MDM2* rs309 polymorphism

The amplification-refractory mutation system polymerase chain reaction (ARMS-PCR) technique was used to study nucleotide changes in the *MDM2* gene. In this technique, the reaction can be performed in a tube. In this study, the ARMS technique was used to determine the presence of the T>G point mutation with two primer pairs (Tables 1 and 2).

**Fig. 1.**
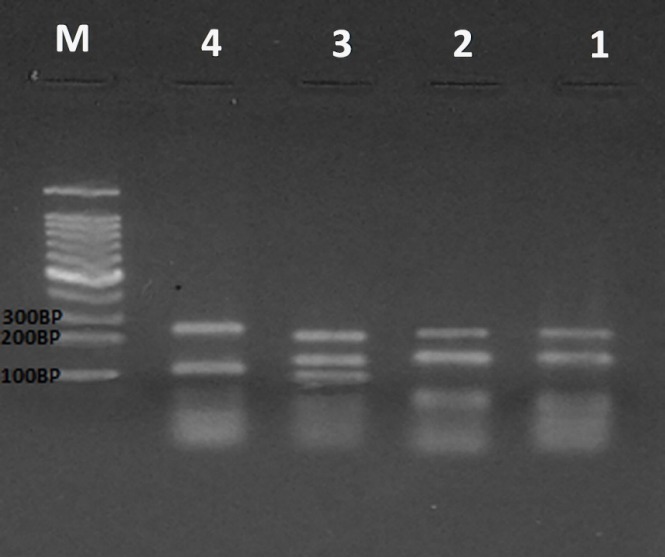
Amplification-refractory mutation system polymerase chain reaction technique of *MDM2* codon 309 polymorphism. Line 1, 224 bands of PCR accuracy and 158 bands of homozygous dominant (GG); line 2, 224 bands PCR accuracy and 158 homozygous dominant (GG) bands; line 3, 224 bands PCR 122 and 158 heterozygous TG bands; line 4, 224 bands PCR accuracy and 122 TT homozygous recessive bands; line M, molecular marker 100 bp.

A proliferation fragment of 224 bp should be seen in all samples as an indicator of the accuracy of PCR. The expected results of ARMS-PCR in this study included the normal genotype (TT), as well as the TG and GG mutant genotypes. After PCR, the products were separated on an agarose gel by electrophoresis and then bands were observed using ultraviolet visualization (Fig. 1).

### Statistical analysis

The statistical analysis was conducted using SPSS version 22 (IBM Corp., Armonk, NY, USA) and p-values of ˂0.05 were considered to indicate statistical significance.

## Results

The characteristics of the participants with ulcerative colitis and the control sample are summarized in Table 2. The mean age of the patients with ulcerative colitis was 46.5 years (range, 28 to 69 years) and that of the control individuals was 45.3 years (range, 26 to 71 years).

The presence of ulcerative colitis was significantly related with age (p < 0.05 [chi-square test]). However, it was not significantly related with sex (p > 0.05 [chi-square test]) (Table 3).

### Association of the *MDM2* polymorphism with the risk of ulcerative colitis

Table 4 shows the allelic frequency of the *MDM2* rs309 polymorphism and the distribution of genotypes. The G allele was present in 48.27% of the ulcerative colitis patients and in 56.09% of the control individuals. The distribution of the *MDM2* genotype in the ulcerative colitis patients was as follows: TT, 37.93%; TG, 27.59%; and GG, 34.48%. This was significantly different from the distribution in the control individuals (GG genotype: 34.48% vs. 43.90%; p < 0.05).

People with the GG phenotype of the *MDM2* gene were more prone to ulcerative colitis (odds ratio, 7.142; 95% confidence interval, 2.400 to 9.542) than those with the TT genotype. The heterozygous genotype of this polymorphism did not show a clear relationship with the risk of ulcerative colitis, but we could nonetheless identify the G allele as risky (Table 4).

## Discussion

In 2005, Sotamaa et al. [13] conducted a study on the *MDM2* gene polymorphism at rs309 in patients with intestinal cancer that included 93 patients and 100 controls. The allelic frequencies of polymorphisms in the patients and control individuals showed Hardy-Weinberg equilibrium, and there was no significant relationship between age and occurrence of this polymorphism [13].

In our study, the polymorphism at this locus was investigated using ARMS-PCR, and a significant relationship was found between the presence of the GG genotype and the incidence of ulcerative colitis disease. A significant relationship was also found between age and ulcerative colitis , but no significant relationship was found for sex.

In 2014 study by Enokida et al. [14] on the rs309 *MDM2* gene polymorphism in lung cancer, the distribution of genotypes showed no significant difference between lung cancer patients and controls (patients: TT, 20.1%; TG, 49.7%; and GG, 30.2%; controls: TT, 21.7%; TG, 47.9%; and GG, 30.4%).

In our study, we found that there was a significant relationship between age and genotype, our results were inconsistent with some previous studies. The distribution of the *MDM2* genotype in ulcerative colitis patients was as follows: TT, 37.93%; TG, 27.59%; and GG, 34.48%. This distribution was significantly different from that observed in controls (TT, 31.70%; TG, 24.40%; and GG, 43.90%).

Mutations in the *P53* gene have been identified in most human cancers, as well as in its downstream signaling pathways, which are mediated by the *P21* and *MDM2* genes; therefore, proper functioning of all three genes is important for the normal function of cells. Consequently, when mutations in any of these genes disrupt critical signaling pathways, they can result in malignancies in human cells [11].

Many studies have found *MDM2* gene mutations in the intestinal system to be associated with cancer [15]. In the current study, the overall aim was to investigate the polymorphism of this gene at rs309 and its association with ulcerative colitis, but a secondary goal was to explore the association between this polymorphism and the risk of cancer.

Since the distribution of the *MDM2* polymorphism in individuals with ulcerative colitis was approximately the same as, it can be concluded that ulcerative colitis is a precedes for the development of ulcers into malignancies.

In this study, it was found that the T>G polymorphism at the rs309 locus of the *MDM2* gene was associated with ulcerative colitis through a statistical analysis. A direct relationship was found between age and ulcerative colitis, while no relationship was found for sex.

Since this gene is directly associated with carcinogenesis (mutation and loss of function), it can be concluded that the occurrence of intestinal inflammation and subsequent ulceration lays the groundwork for subsequent cancer.

## Figures and Tables

**Table 1.** The sequences of the primers used in this study

Primer name	Direction	Primer sequences
*MDM2*(M)	Forward (1)	5´ GGGGGCCGGGGGCTG CGG GGC CGT TT 3´
*MDM2*(M)	Reverse (1)	5´ TGC CCACTG AAC CGG CCC AAT CCC…CAG 3´
*MDM2*(C)	Forward (2)	5´ GGC AGT CGC CGC CAG GGA GCA GGG CGG 3´
*MDM2*(C)	Reverse (2)	5´ ACC TGC CAT CAT CCG GAC CTC CCG…TGC 3´

**Table 2.** Thermocycler program for *MDM2* gene amplification

No.	Stage	Temperature (℃)	Time
1	Initial denaturation	95	15 min
2	Denaturation	95	45 s
3	Annealing	64	45 s
4	Extension	72	1 min
5	Final extension	72	7 min
Cycles (2-4)	35		

**Table 3.** Demographic characteristics of patients with ulcerative colitis and controls

Characteristic	Case	Control	χ^2^
Total (n=256)	174	82	
Age (yr)			
≤50	110 (63.2)	48 (58.5)	0.006
>50	64 (36.8)	34 (41.5)	
Sex			
Male	78 (44.8)	28 (34.1)	0.4
Female	96 (55.2)	54 (65.9)	

Values are presented as number (%).

**Table 4.** Genotype and allele frequency of MDM2 in patients with ulcerative colitis and controls

Genotype	Case (n = 174)	Control (n = 82)	OR (95% CI)
*MDM2* codon 309			7.142 (2.400-9.542)
TT	66 (38.0)	26 (31.7)	
TG	48 (27.6)	20 (24.4)	
GG	60 (34.5)	36 (43.9)	
G allele frequency	48.27	56.09	

OR, odds ratio; CI, confidence interval.
